# Rust-Layer Characteristics and Pit Morphology on the Inner and Outer Surfaces of a Retired Q355C Hydropower Penstock

**DOI:** 10.3390/ma19214616

**Published:** 2026-10-30

**Authors:** Tao Wu, Jiudao Zhou, Taiwen You, Linchanghong Shi, Mingzhi Chen, Chi Zhang, Xiaojia Yang, Yuping He

**Affiliations:** 1 Goupitan Power Plant, Guizhou Wujiang Hydropower Development Co., Ltd., Guiyang, 550002, China; 2 Haidai Campus, Institute for Advanced Materials and Technology, University of Science and Technology Beijing, Beijing 100083, China; yangxiaojia@ustb.edu.cn

**Keywords:** penstock steel, rust layer, pit morphology, galvanic current monitoring, electrochemical impedance spectroscopy

## Abstract

A hydropower penstock corrodes on two faces that share one steel wall but almost nothing else, because the inner surface stays permanently wetted by flowing reservoir water while the outer surface is wetted only intermittently by the plant atmosphere. The two surfaces of a service-retired Q355C penstock were compared using cross-sectional electron microscopy, potentiodynamic polarisation, impedance spectroscopy, confocal profilometry of the descaled surfaces and 720 h of continuous galvanic-current monitoring sampled once per minute. The outer rust layer was thicker and exhibited cracks, delamination, and pores, while its fitted rust-layer resistance was lower than that of the inner surface. The laboratory electrochemical measurements did not establish a reliable comparison of service corrosion rates between the two surfaces. One of the 31 outer-surface pits exceeded a depth-to-equivalent-mouth-diameter ratio of 0.5, whereas none of the 59 inner-surface pits did; however, the proportion difference was not statistically significant. The available 720 h galvanic-current records showed overall decreases on both surfaces, with temporal fluctuations.

## 1. Introduction

The penstock is the load-bearing conduit that delivers water from the reservoir to the turbine of a hydropower station, and its structural integrity governs both generation availability and plant safety [1,2]. Because a penstock is a thin-walled pressure structure, corrosion damage translates almost immediately into loss of load-bearing section. General thinning erodes the design safety margin, while localised attack introduces stress concentrators that can initiate fatigue or brittle fracture long before the mean wall thickness reaches its allowable limit [2,3]. Corrosion control of penstocks has therefore become a recurrent concern in the operation of ageing hydropower assets, many of which are now well beyond half of their nominal design life [3,4].

What makes a penstock unusual among corroding structures is that a single steel wall is exposed to two entirely different environments at the same time. The inner surface is permanently immersed in flowing reservoir water and is additionally subject to hydrodynamic shear and, in sediment-laden rivers, to particle impingement, so the damage it accumulates is usually described as a combination of general corrosion, pitting and erosion-corrosion [1]. Flow does not simply accelerate an otherwise uniform process. The flow-accelerated corrosion of low-alloy steel in chloride solution depends jointly on the hydrodynamic condition and on the chemistry of the steel [5], and electrode arrays operated under flowing electrolyte show that anodic and cathodic areas redistribute across a surface as the flow field changes [6]. Field inspections repeatedly show that once the internal protective coating is breached, direct contact between the steel and the water accelerates attack sharply [4]. The outer surface faces the gallery or open-air environment of the plant, where corrosion is controlled by atmospheric exposure, condensation, seepage, and the condition of the external coating [1]. Intermittent wetting is a qualitatively different regime rather than a milder form of immersion, and the corrosion of pipeline steel under alternating wet and dry periods depends on the duration of each period as much as on the flow conditions during the wet phase [7]. Wet-dry cycling in chloride-bearing conditions also selects which rust phases form, favouring akaganeite [8], and atmospheric sulfur species further modify the particles produced in a chloride environment [9].

The layer that forms under each of these regimes is what actually mediates the corrosion that follows, and its structure rather than its mass is what matters. Detailed characterisation of weathering-steel rust grown in high-chloride conditions reveals a layered architecture whose compactness varies over a few micrometres [10], and chloride ions taken up into the product film of a Cr-Ni steel alter its protectiveness during tropical marine exposure [11]. Much of the effort devoted to improving low-alloy steels has consequently been aimed at controlling the rust rather than the substrate. Grain refinement changes rust nucleation and the corrosion resistance that results [12]; calcium and magnesium treatment changes the inclusion population, and with it the sites at which localised attack begins [13]; silicon is essential to the resistance of bridge steel in chloride atmospheres [14], and niobium and antimony improve resistance to stress corrosion cracking in polluted marine air [15]. The rust is not a passive barrier either. Corrosion products formed under wet-dry cycling sustain their own cathodic reaction, and the alloy content of the steel governs how readily they do so [16].

Separating the barrier contributed by such a layer from the charge-transfer process beneath it calls for a method sensitive to both, and impedance spectroscopy combined with surface analysis has been used in exactly this way to follow corrosion product layers on low-alloy steel [17]. Where the layer is laterally heterogeneous the consequences are severe. At a waterline, where one part of a steel surface is immersed, and an adjoining part is not, the rust layer itself controls where localised corrosion starts and how far it propagates [18]. A penstock presents the same asymmetry in a more extreme form, with the two environments separated by the wall rather than by a free surface.

Existing work on penstocks is nonetheless dominated by inspection practice, safety assessment and protective strategy [1,2,3], and a substantial literature now exists on the causes of internal corrosion in particular [4]. What is largely missing is a direct, like-for-like comparison of the two surfaces of the same pipe. This is not a trivial omission. Comparisons drawn between different structures, or between a field surface and a laboratory coupon, inevitably confound the effect of the environment with differences in steel chemistry, thermomechanical history, weld position and service duration. Only a single retired component carries both environments on one substrate, and only such a component allows the environmental contribution to be isolated.

At the same time, the way corrosion evidence is collected has changed. Conventional field assessment relies on periodic coupon retrieval or on wall-thickness surveys performed during scheduled outages, which give a handful of time points and average over the whole exposed area. Sensor-based continuous monitoring, in which a galvanic couple of the structural steel against a noble counter-electrode is polled at minute resolution and streamed to a database, resolves behaviour that discrete sampling cannot see [19,20]. Electrical resistance probes have tracked rust growth on weathering steel in the same continuous manner [21], and combined outdoor exposure with on-site monitoring has been applied to reinforcing steel in the plateau environment of western China [22]. The volume of data such systems generate, of the order of 10^5^ to 10^6^ records per sensor per year, has motivated the description of this approach as corrosion big data. It has supported statistical treatment of the galvanic current record itself [23] as well as machine-learning models of atmospheric corrosion kinetics that carry the evolving rust layer as a state variable [24,25].

The same shift is visible on the damage side. Three-dimensional surface metrology combined with image processing now makes it practical to describe a whole population of pits statistically [26], and clustering of measured pit depths on buried cast iron has been used to separate populations that a single maximum value would merge [27]. This matters because it is the population, and specifically its deepest members, rather than the average that determines the risk of through-wall penetration.

The present work brings these developments to bear on the penstock problem. A Q355C penstock retired from service at a hydropower station was sectioned so that its inner and outer surfaces could be characterised in parallel, and four independent kinds of evidence were assembled on the same material. These were the architecture and composition of the rust layer in cross-section, the steady-state and impedance response of the rusted surfaces, the statistical distribution of pit shape after descaling, and a 720 h continuous galvanic-current record sampled once per minute that comprises 4.32 × 10^4^ points per surface. The aim is not merely to establish which surface corrodes faster, a question that turns out to have a less interesting answer than expected, but to determine which physical property of the rust layer discriminates the two surfaces and how that property controls the shape of the damage each surface accumulates.

## 2. Experimental

### 2.1. Material and Specimen Preparation

The material was taken from a service-retired penstock segment of a hydropower station. The pipe substrate is Q355C low-alloy structural steel conforming to GB/T 1591. In accordance with SL/T 432-2024 (Chinese water conservancy industry standard) [28], this grade is the dominant material for penstock manufacturing in China, and its chemical composition is presented in Table 1. The residual levels of Cr, Ni and Cu are all at or below 0.02 wt%, so the steel carries none of the deliberate weathering additions that stabilise a protective rust layer on weathering grades. Detailed field service parameters can be found in Supplementary Tables S1 and S2.

Specimens were cut from the intact pipe wall so that the as-retired corrosion state of both faces was preserved. The inner and outer surfaces of every specimen therefore originate from the same heat, the same rolling history and the same service period, and differ only in the environment to which they were exposed. For microstructural examination, coupons of 10 mm × 10 mm × 4 mm were removed by wire electrical discharge machining, ground successively on 400#, 800#, 1500# and 2000# SiC papers, and mirror-polished with 2.5 μm, 1.5 μm and 0.5 μm SiO_2_ suspensions before cleaning in ethanol and air drying. Specimens intended for electrochemical testing and for rust-layer cross-sectioning were not ground on the exposed face, so that the service-formed rust remained intact. For each measurement type and each surface condition, three independent replicate specimens were tested. One measurement was performed on each specimen, and the reported mean was calculated from the three specimen results

### 2.2. Microstructural Characterisation

Polished coupons were etched for 10 s in 4 vol.% nital and examined by optical microscopy and scanning electron microscopy.

### 2.3. Rust-Layer Cross-Section and Elemental Mapping

Rusted specimens were mounted in epoxy resin, and the mount was ground on SiC paper until the rust and steel cross-section was exposed, then polished. Cross-sections were imaged in backscattered-electron mode on an FEI scanning electron microscope (FEI Company, Hillsboro, OR, USA) equipped with an energy-dispersive X-ray spectrometer, and elemental distributions of Fe, Cr, Ni, O, S, and Cl were mapped across the rust layer and into the substrate. Rust-layer thickness was taken as the mean separation between the outer rust boundary and the rust and steel interface over the imaged field.

### 2.4. Electrochemical Measurements

Electrochemical tests were performed on the as-retained rusted surfaces in a conventional three-electrode cell using a Donghua DH7006 workstation (Donghua Analysis Instruments Co., Ltd., Jingjiang, China), with the rusted specimen as the working electrode, a platinum plate as the counter electrode, and a saturated calomel electrode as the reference. The electrolyte was 3.5 wt.% NaCl at room temperature. It is used as a standard aggressive reference electrolyte so that the two rust layers are interrogated under identical, well-defined and sufficiently conductive conditions, which is the usual practice when the object of comparison is the rust layer rather than the service environment.

The open-circuit potential was recorded for 1 h before each measurement to allow the rusted interface to stabilise. Electrochemical impedance spectroscopy was measured at the open-circuit potential from 100 kHz to 10 mHz with a 10 mV perturbation, and the impedance spectra were fitted using an equivalent circuit with the configuration *R*_s_(*Q*_f_(*R*_f_(*Q*_dl_*R*_ct_))), in which *R*_s_ is the solution resistance, *R*_f_ and *Q*_f_ describe the rust layer, and *R*_ct_ and *Q*_dl_ describe charge transfer and the electrical double layer at the rust and steel interface. Constant-phase elements were used in place of ideal capacitors to account for the capacitance dispersion introduced by the roughness and porosity of a service-formed rust layer. Potentiodynamic polarisation was measured on a separate specimen from 300 mV below to 300 mV above the open-circuit potential at 0.333 mV s^−1^. Electrochemical parameters are reported as mean ± standard deviation across three independent specimens for each surface condition. For EIS, each specimen spectrum was fitted separately using the same equivalent circuit, and the resulting parameter estimates were subsequently summarised.

The polarization data were fitted using the full Butler–Volmer expression in Equation (1) over a potential window of ±60 mV relative to the corrosion potential. The iR correction used the solution resistance obtained from impedance measurements on a separate specimen. Its applicability to the polarization specimen was not independently verified, introducing uncertainty into the corrected potentials and fitted parameters. Because cathodic rust reduction may contribute alongside oxygen reduction, the fitted parameters are treated as apparent estimates.

### 2.5. Damage Morphology and Pit Statistics After Descaling

Corrosion products were removed chemically, and the descaled surfaces were characterised by laser scanning confocal microscopy. Corrosion products were removed using a hydrochloric acid cleaning solution prepared according to GB/T 16545-2015, consisting of 500 mL HCl, 500 mL H_2_O, and 3.5 g hexamethylenetetramine (C_6_H_12_N_4_). Cleaning was performed in an ultrasonic bath maintained at 25 °C for 5 min. The specimens were subsequently rinsed with deionized water and ethanol, blown dry, and further dried in an oven. No blank correction was performed. Two descriptors were extracted. The arithmetical mean height *S*_a_ quantifies the mean deviation of the surface from its mean plane, so that a small *S*_a_ indicates a surface that has been attacked uniformly [12]. The pit shape factor *K* is defined as the ratio of the maximum depth of a pit to its mouth diameter [22,26,29,30]. A hemispherical pit corresponds to *K* = 0.5, so *K* below 0.5 describes a pit that has spread laterally more than it has penetrated, and *K* above 0.5 describes a pit that has propagated preferentially into the substrate. Surface depressions were identified automatically using the instrument’s built-in software(KEYENCE VK-X Series 1.1.20.83). A reference plane was fitted by least squares over the entire scanned field to remove specimen tilt and was assigned a height of 0 μm. Filtering was applied to suppress isolated noise spikes. Pixels below this plane were classified as depressions, and regions containing fewer than 100 pixels were excluded. Pit depth, h, was measured from the fitted plane to the deepest point of each retained pit. Mouth diameter was calculated as the equivalent-circle diameter of the projected depression area. The analysed area was 6634 μm × 4737 μm (31.425 mm^2^) for each surface. Coalesced depressions were excluded from quantitative measurements of individual pit depth and mouth diameter and from pit-density counts because their individual mouth boundaries could not be reliably defined. The resulting dataset contained 59 inner-surface pits and 31 outer-surface pits, corresponding to individual-pit densities of 1.877 and 0.986 pits mm^−2^, respectively. These statistics describe the retained individual pits. *K* was presented as a frequency distribution to describe the variability in pit geometry. Skewness and excess kurtosis were calculated from standardized central moments without small-sample bias correction. For each surface, 100,000 bootstrap samples were generated by resampling individual pits with replacement, and 95% confidence intervals were obtained using the percentile method. A sensitivity analysis excluded the largest outer-surface K value (0.83149), while the complete dataset was retained for the primary analysis. The proportions of pits with K > 0.5 were compared using a two-sided Fisher’s exact test.

### 2.6. Continuous Galvanic-Current Monitoring

**Figure 1:**
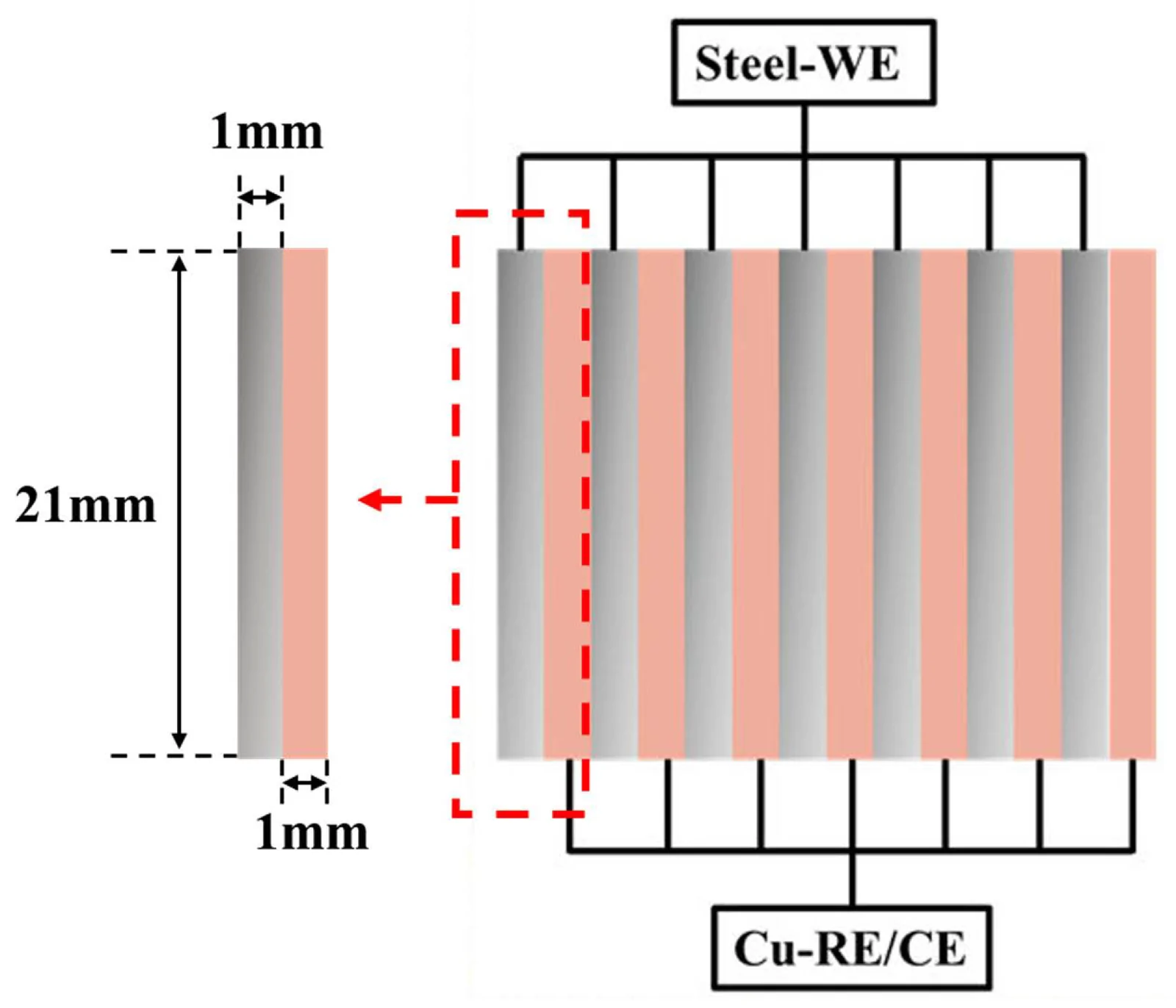
Schematic of the galvanic-couple corrosion monitoring sensor.

Continuous monitoring used a galvanic-couple sensor consisting of seven interdigitated pairs of bare fresh Q355C steel (no pre-existing service-aged rust) and copper. The exposed area of each pair was 21 × 2 mm^2^, and the total exposed area was 294 mm^2^. It should be highlighted that this sensor monitors the evolution of newly formed rust under field environment; it does not directly reproduce dynamic behaviour of thick service-generated rust layers on retired penstock wall specimens, shown schematically in Figure 1. The penstock steel serves as the working electrode, the copper electrode acts simultaneously as counter and pseudo-reference electrode, and the two metals in each pair are separated by a thin insulating spacer. The galvanic current flowing between the steel and the copper array is governed by the ease with which the anodic dissolution of the steel can proceed, and therefore tracks the instantaneous corrosion activity of the steel surface.

The sensor assembly was installed in the service environment, and the acquisition unit recorded the galvanic current once per minute and transmitted the record over a 4G link to a cloud database. Monitoring ran continuously for 720 h, giving 4.32 × 10^4^ samples per surface and 8.64 × 10^4^ samples in total. Non-overlapping hourly means were calculated from 60 one-minute observations, yielding 720 values per surface. Spearman’s correlation described the association with time. Linear-trend confidence intervals and two-sided *p*-values were estimated using a circular block bootstrap of detrended residuals (72 h blocks; 100,000 resamples), assuming approximately stationary residuals. Alternative block lengths and daily means were used for sensitivity checks. Time points were not treated as independent specimen replicates.

The resulting time series is displayed as a corrosion clock plot. In this representation, each concentric ring carries the 1440 samples of one 24 h period; the angular coordinate is time of day, and the radial coordinate is elapsed exposure time, with the outermost ring corresponding to the start of monitoring and the innermost ring to its end. The advantage of the representation is that it separates the diurnal cycle, which appears as angular structure, from the secular trend of the corrosion process, which appears as a radial gradient. Both are difficult to read from a conventional current-against-time trace of the same length. The colour scale spans 16.00–18.00 μA in eight bands of 0.25 μA.

## 3. Results

### 3.1. Substrate Microstructure

**Figure 2:**
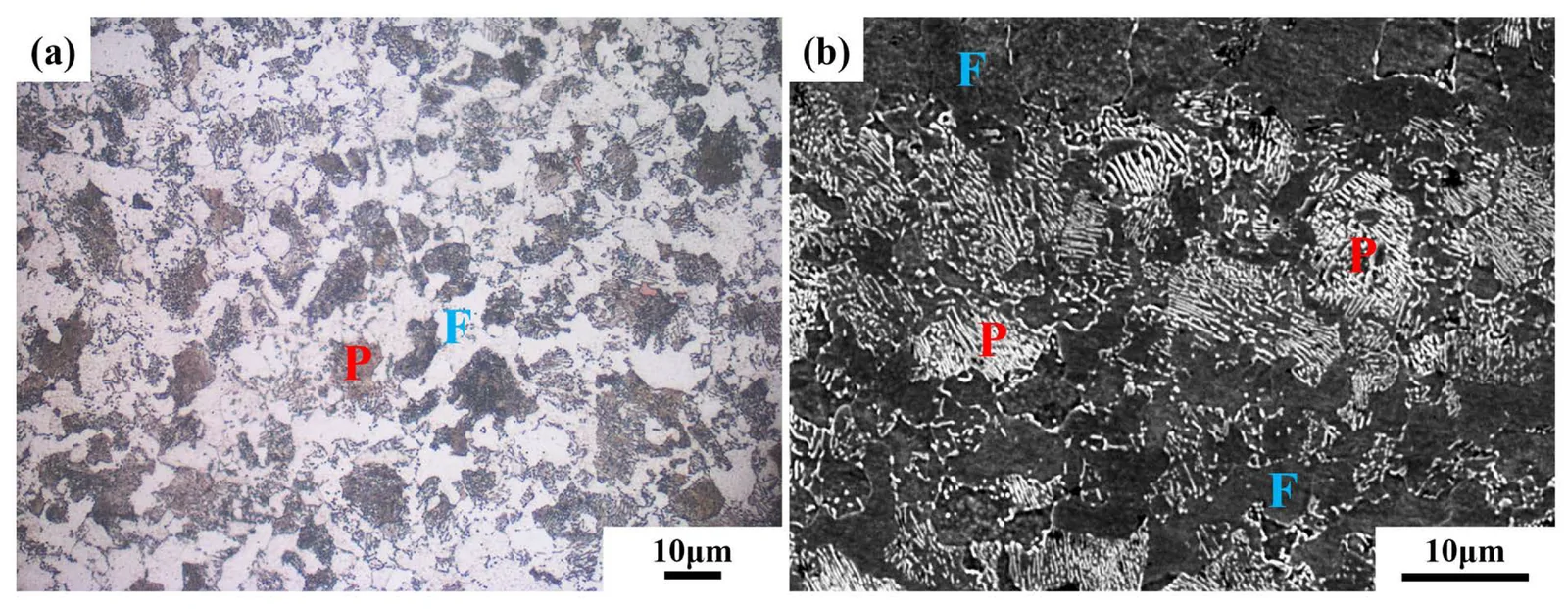
Microstructure of the Q355C penstock steel. (**a**) Optical micrograph. (**b**) SEM micrograph. (P: pearlite, F: ferrite)

Figure 2 shows the microstructure of the pipe body. The optical micrograph in Figure 2a reveals a two-phase structure in which bright ferrite forms the matrix and darker pearlite is distributed through it as discrete colonies, the microstructure expected of a hot-rolled low-alloy structural steel of this grade. At higher magnification in Figure 2b, the pearlite colonies resolve into a regular alternation of ferrite and cementite lamellae; the lamellar structure is intact, and the phase boundaries are sharp. No decarburised layer, banding, or abnormal grain structure was observed. Figure 2 shows the bulk ferrite–pearlite microstructure. It does not separately characterize the near-surface regions of the inner and outer faces; therefore, possible microstructural differences between these regions cannot be excluded.

### 3.2. Rust-Layer Architecture and Elemental Distribution

**Figure 3:**
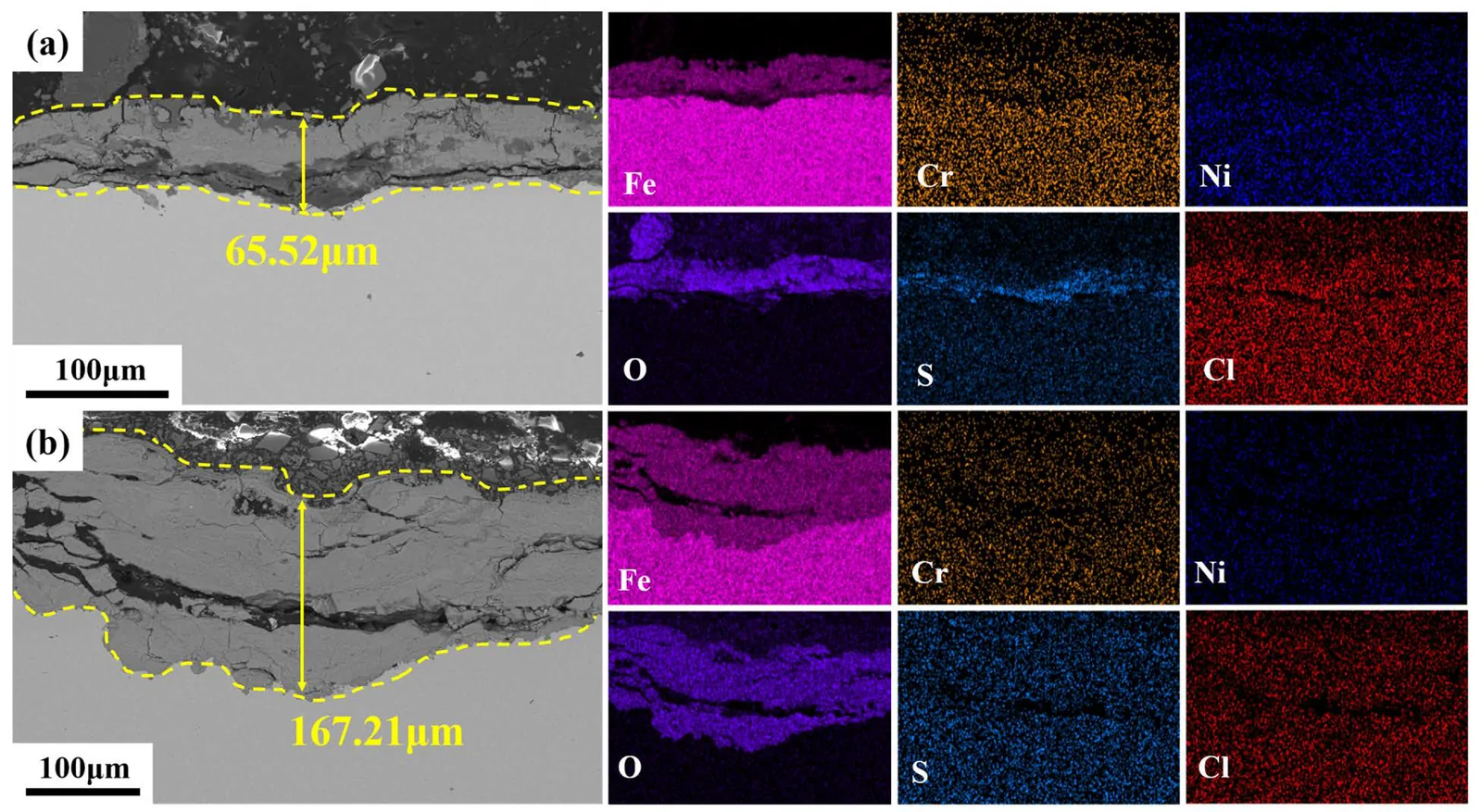
Cross-sectional backscattered-electron images and EDS elemental maps of the rust layers. (**a**) Inner surface. (**b**) Outer surface.

Figure 3 shows one of the four measured fields for each surface, whereas Table 2 summarizes the thickness measurements across all four fields. The two layers differ in thickness and, more importantly, in structure.

The inner-surface rust in Figure 3a is 65.52 μm thick on average. It is continuous, adheres closely to the substrate, and contains few internal defects. Cracking is limited; there is no evidence of interlayer separation, and the rust and steel interface is gently undulating rather than deeply indented.

The outer-surface rust in Figure 3b reaches 167.21 μm, some 2.6 times the inner value, but is structurally far inferior. It is traversed by long cracks running parallel to the substrate, by transverse cracks linking them, and by extensive interlayer delamination and porosity. Several of these defects extend from the outer boundary of the rust to within a short distance of the steel, so the layer does not form a continuous barrier despite its thickness. The rust and steel interface is correspondingly more irregular than on the inner surface.

The elemental maps show Fe and O rich corrosion products in both layers. These observations are consistent with iron-containing corrosion products but do not identify their crystalline phases or distinguish specific oxides from oxyhydroxides. Oxygen is concentrated in a well-defined band coincident with the inner rust of Figure 3a, whereas in Figure 3b the oxygen signal is distributed across the full thickness of the layer and is interrupted by the crack network. Sulfur signal can be detected in both rust layers. For the outer surface, this may relate to atmospheric sulfur species; for the immersed inner surface, potential sulfur sources include dissolution of MnS inclusions in the steel substrate, water-borne sulfate, and microbially generated sulfur-containing species [9]. Cl distribution is heterogeneous within the outer-surface rust layer, while Cl intensity stays close to detection noise for inner-surface rust. The Cr and Ni maps of both surfaces sit at the noise level of the technique, which is the expected result given the bulk residual contents of 0.02 wt% Cr and 0.01 wt% Ni recorded in Table 1. No conclusion about the partitioning of these elements between rust and substrate can be drawn from the maps, and none is drawn here.

### 3.3. Electrochemical Response of the Rusted Surfaces

**Figure 4:**
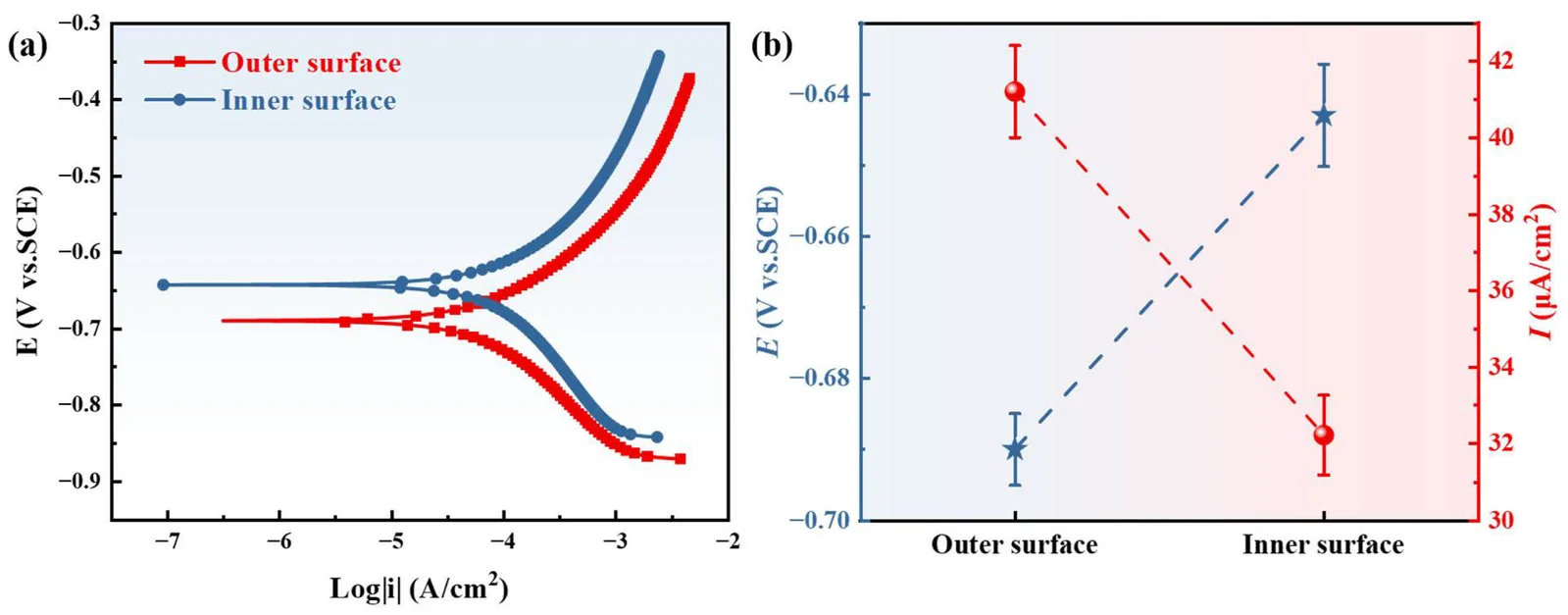
Potentiodynamic polarisation behaviour in 3.5 wt.% NaCl. (**a**) Polarisation curves. (**b**) Fitted corrosion potential and corrosion current density.

Figure 4a shows potentiodynamic polarisation curves for the two rusted surfaces, and Figure 4b compares the fitted parameters, which are listed in Table 3. It is −0.643 V against the saturated calomel electrode for the inner surface and −0.690 V for the outer surface, so the inner surface exhibits a 47 mV more positive mixed potential, determined jointly by anodic metal dissolution and multiple cathodic processes including oxygen reduction and possible rust-phase reduction [13,14].

Neither branch of either curve possesses an extended region of constant slope. On the anodic branch, the local slope rises monotonically with current, from about 70 mV per decade at 40 mV above the corrosion potential to more than 300 mV per decade at 250 mV above it, which is the signature of a dissolution reaction increasingly limited by transport through the rust layer. On the cathodic branch, the slope passes through a maximum near 100 mV below the corrosion potential and falls again at more negative potentials, as oxygen reduction approaches its diffusion-limited plateau and a second cathodic reaction begins. Straight-line Tafel extrapolation from an arbitrarily chosen segment is therefore not defensible on these data, and the kinetic parameters in Table 3 were obtained instead by the ohmic-corrected Butler-Volmer fit described in Section 2.4.

**Figure 5:**
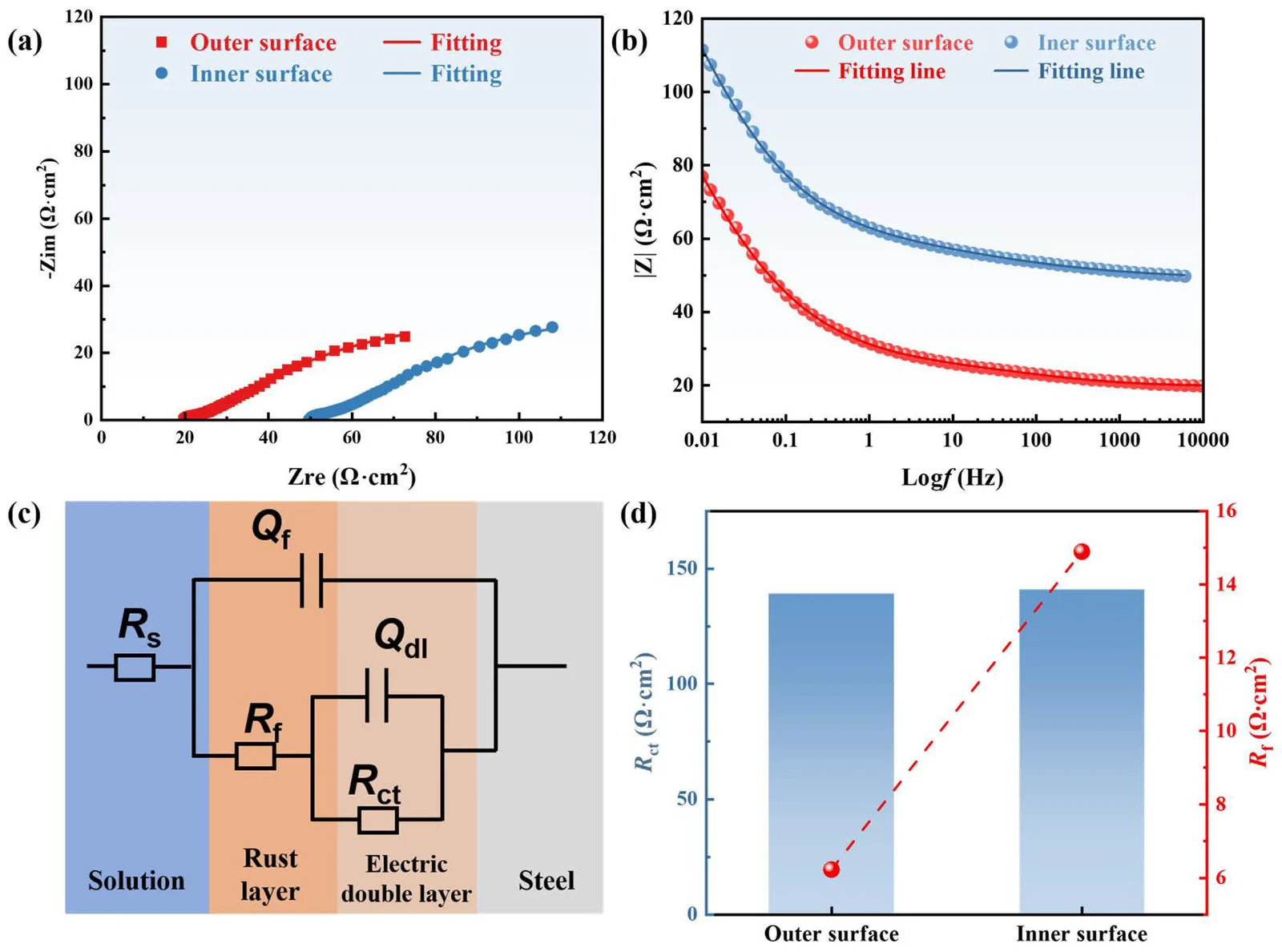
Electrochemical impedance response in 3.5 wt.% NaCl. (**a**) Nyquist plots. (**b**) Bode magnitude plots. (**c**) Equivalent circuit. (**d**) Fitted charge-transfer and rust-layer resistances.

The revised apparent corrosion-current densities were 32.23 μA/cm^2^ for the inner surface and 41.21 μA/cm^2^ for the outer surface. These values characterize the response in the reference electrolyte and should not be interpreted directly as service corrosion rates. Parameters derived from the same Butler–Volmer fit do not provide independent mutual validation. Figure 5 presents the impedance response and Table 4 lists the fitted parameters. The Nyquist spectra show broad, overlapping responses rather than clearly separated semicircles. A small high-frequency arc-like feature is discernible in the outer-surface spectrum. At the lowest measured frequency, the real impedances were approximately 108 Ω·cm^2^ and 72.7 Ω·cm^2^ for the inner and outer surfaces, respectively. Neither arc is closed at 10 mHz, therefore, all fitted resistance values rely on low-frequency extrapolation and carry notable fitting uncertainty. Fitted absolute resistances should not be treated as directly measured true values. It should be noted further that a ratio between two lower-bound fitted quantities is not guaranteed to represent a lower-bound estimate. In the Bode magnitude plot of Figure 5b, the modulus of the inner-surface specimen exceeds that of the outer-surface specimen at every frequency, reaching 111.5 Ω cm^2^ at the lowest frequency against 76.9 Ω cm^2^.

That comparison must be made with care. The high-frequency intercepts of the two spectra differ by a factor of 2.5, 49.7 Ω cm^2^ for the inner-surface cell against 19.7 Ω cm^2^ for the outer-surface cell, although both specimens were measured in the same electrolyte. The difference in solution resistance indicates differences in the effective ohmic measurement conditions, but does not uniquely identify unequal exposed areas. Although (*R*_f_/*R*_ct_) cancels a common area-normalisation factor, it does not eliminate possible effects of non-uniform current distribution or differences in effective interfacial areas

Fitting with the circuit of Figure 5c gives the parameters compared in Figure 5d. The fitted *R*_ct_ values were 140.7 and 138.9 Ω cm^2^ for the inner and outer surfaces, with approximate 95% confidence intervals of 118.8–162.6 and 121.9–155.9 Ω cm^2^, respectively. Their numerical proximity does not establish identical intrinsic charge-transfer kinetics, particularly given the dependence on low-frequency extrapolation and effective electrochemical area. The rust-layer resistance differs by a factor of 2.4, 14.8 Ω cm^2^ against 6.21 Ω cm^2^. The ratios *R*_f_/*R*_ct_ were 0.105 and 0.045 for the inner and outer surfaces, respectively. The corresponding fractions of total interfacial resistance, *R*_f_/(*R*_f_ + *R*_ct_), were 9.5% and 4.3%. These ratios cancel a common geometric-area normalization factor but may still be affected by differences in effective electrochemical area. No lower-bound interpretation is assigned to the fitted resistance ratios. Even if individual quantities were lower bounds, their ratio would not generally constitute a lower bound on the corresponding true ratio. The *Q*_dl_ values are CPE coefficients rather than directly measured double-layer capacitances. With *n*_dl_ = 0.538 and 0.478, the responses are strongly non-ideal; *Q*_dl_ cannot be directly interpreted in F cm^−2^ or used alone to infer active surface area. Its magnitude may reflect distributed interfacial responses and model-dependent parameter allocation.

### 3.4. Damage Morphology and Pit-Shape Statistics

**Figure 6:**
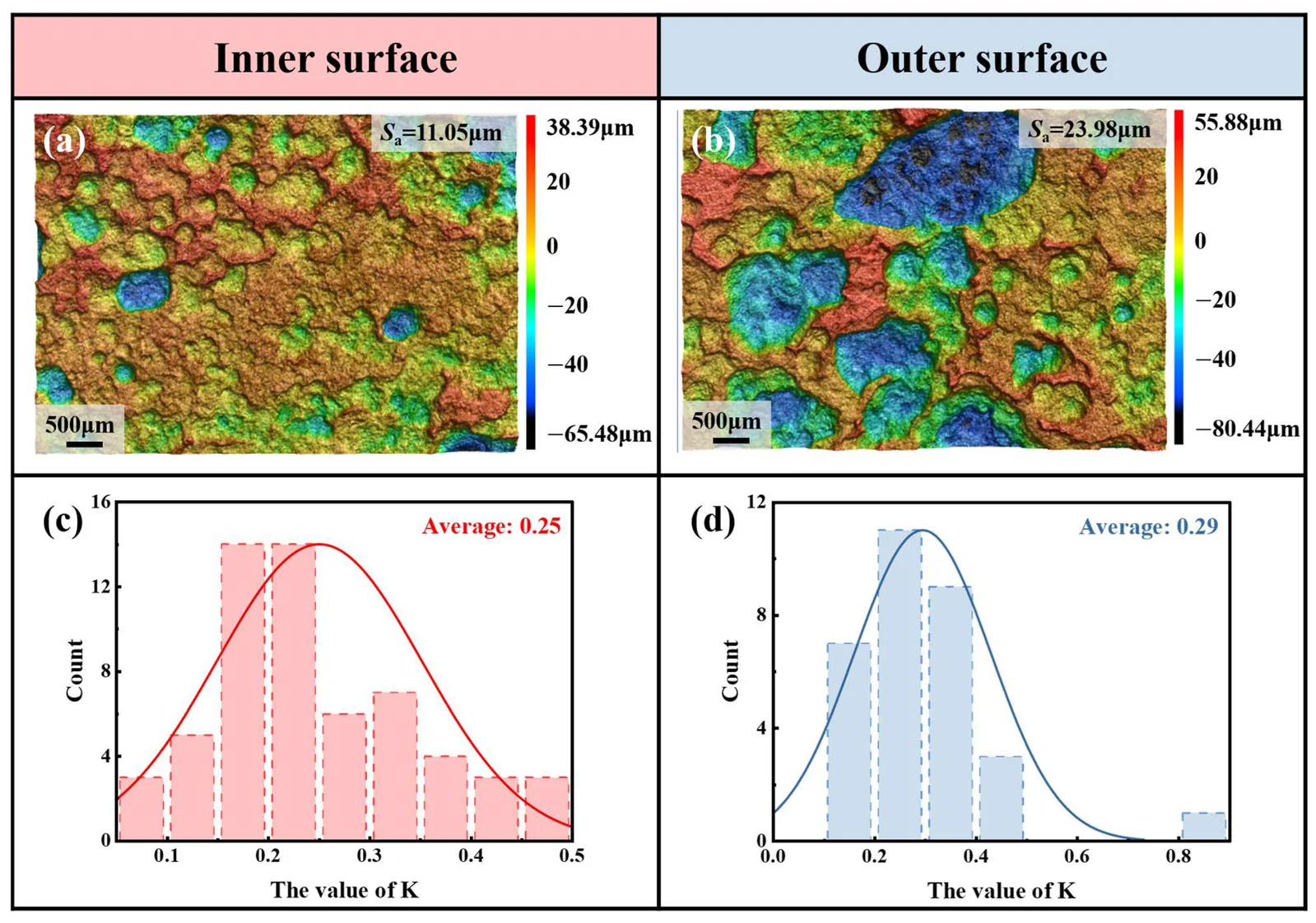
Damage morphology after descaling and pit shape factor distributions. (**a**,**c**) Inner surface. (**b**,**d**) Outer surface.

Figure 6 shows the descaled surfaces and the corresponding distributions of the pit shape factor, and Table 5 collects the quantitative descriptors obtained from 59 pits on the inner surface and 31 pits on the outer surface. The skewness and excess kurtosis of the pit shape factor K, together with their bootstrap 95% confidence intervals and the sensitivity analysis excluding the pit with K = 0.83, are summarised in Table 6

Both surfaces carry a dense population of pits of varying size, but the character of the damage differs. The inner surface in Figure 6a has an arithmetic mean height of 11.05 μm and a maximum pit depth of 65.48 μm. The original measurement records were checked, confirming that the maximum inner-surface pit depth of 65.48 μm and the mean inner rust thickness of 65.52 μm in the field shown in Figure 3a were obtained independently by surface profilometry and cross-sectional microscopy, respectively. Their numerical proximity is coincidental and does not indicate a one-to-one relationship between pit depth and rust thickness. The outer surface in Figure 6b has 23.98 μm and 80.44 μm. The 2.2-fold difference in *S*_a_ is far larger than the 1.2-fold difference in maximum depth, which indicates that the two surfaces differ less in how deep their deepest features are than in how unevenly the attack is distributed across the surface as a whole.

The shape-factor distributions in Figure 6c and d make the same point in terms of pit geometry, and they do so more sharply than the mean values suggest. The mean shape factor is 0.250 on the inner surface and 0.295 on the outer surface, but this difference is not statistically significant, returning *p* = 0.10 by the Mann-Whitney test and *p* = 0.11 by the Welch test. What differs is the shape of the distribution. The inner-surface population is mildly right-skewed, with a skewness of 0.61 and a kurtosis of −0.35, and its maximum value is 0.493. The outer-surface sample had a skewness of 1.934 and an excess kurtosis of 6.349, with bootstrap 95% confidence intervals of −0.537 to 2.551 and −1.268 to 9.322, respectively. After excluding the largest K value (0.83149), these statistics changed to −0.176 and −0.919, indicating that the pronounced right-skewness of the complete sample was strongly influenced by this single extreme pit.

One of the 31 outer-surface pits and none of the 59 inner-surface pits exceeded K = 0.5. The difference in proportions was not statistically significant (two-sided Fisher’s exact test, *p* = 0.344). These observations do not establish a higher population proportion of high-K pits on the outer surface. Not one of the 59 pits measured on the inner surface is deeper than a hemisphere of the same mouth width. On the outer surface, one pit in thirty-one exceeds that limit, and it does so by a wide margin, reaching a depth 0.83 times its own width. Neither distribution is normal, both being rejected by the Shapiro-Wilk test at *p* = 0.028 for the inner surface and *p* = 0.0002 for the outer surface, so the fitted normal curves drawn in Figure 6c,d should be read as guides to the central body of each population rather than as descriptions of its tail. Without corresponding wall-thickness data, the measured maximum pit depths cannot establish through-wall penetration risk. Only one of the 31 outer-surface pits exceeded K = 0.5, so the upper-tail characteristics should be interpreted cautiously. It should be highlighted that the total pit sample size (*n* = 59 for inner surface, *n* = 31 for outer surface) is limited. Although obvious distribution differences and strong right-skewness of the outer-surface pit population can be observed, this dataset cannot support quantitative extreme-value analysis for service-life prediction or penetration risk modelling, which demands a far larger pit sample population.

### 3.5. Continuous Galvanic-Current Records

**Figure 7:**
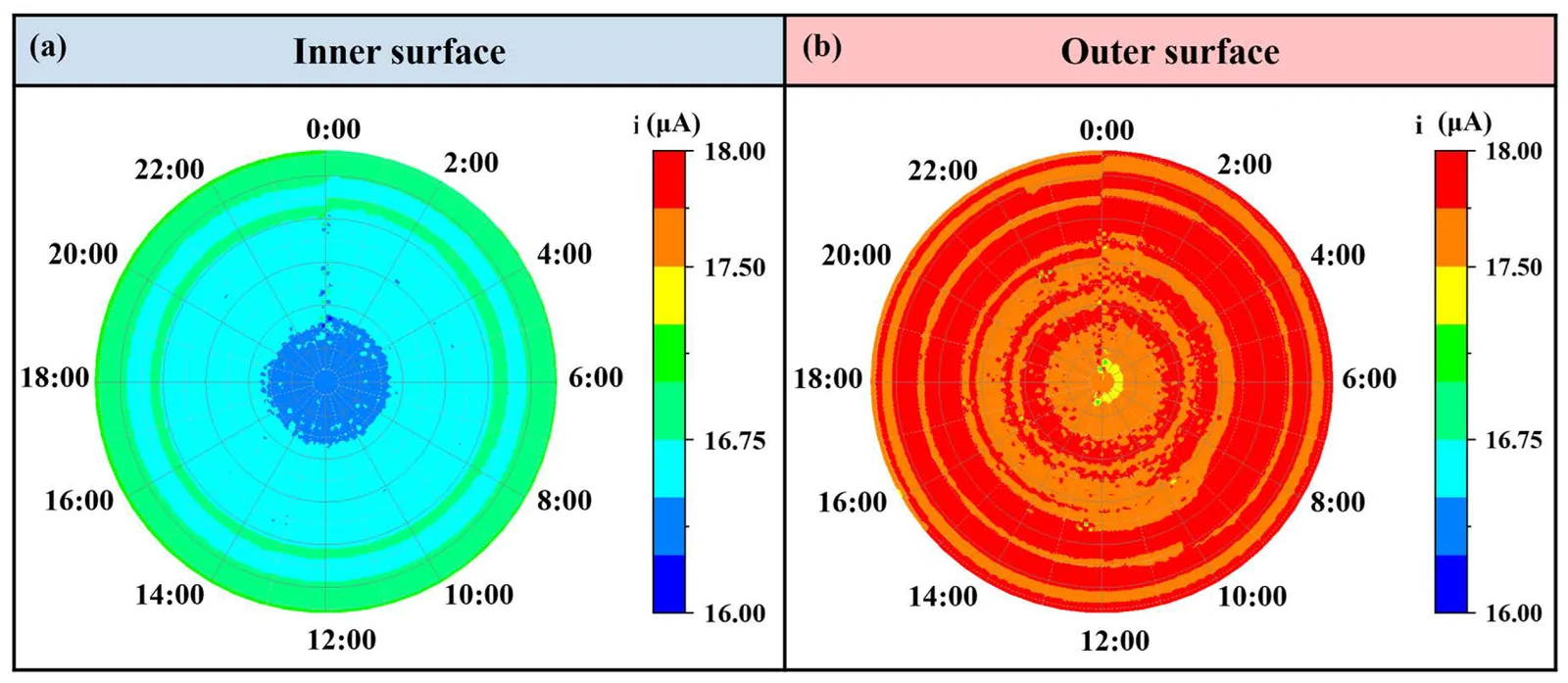
Corrosion clock plots of the galvanic current recorded for 720 h. (**a**) Inner surface. (**b**) Outer surface.

Figure 7 shows the corrosion clock plots assembled from the 720 h monitoring records. The two surfaces differ both in the level of the galvanic current and, more informatively, in how that current evolves.

The inner-surface current in Figure 7a showed an overall decrease during the analysed period, with temporal fluctuations. Ring-averaged values fall from approximately 16.9 μA during the first 72 h to approximately 16.4 μA over the final 72 h, and the record as a whole averages approximately 16.7 μA. More than 90% of the record lies within the two bands spanning 16.50–17.00 μA, and the colour field is smooth, with the bands forming continuous concentric rings rather than patches. The surface behaves as a single, laterally uniform electrode whose activity declines steadily as exposure proceeds.

On the outer surface in Figure 7b, the behaviour is different in kind. The current is approximately 1.1 μA higher throughout, averaging approximately 17.8 μA, and about 60% of the record falls in the highest band, 17.75–18.00 μA. The outer-surface current fluctuated between approximately 17.5 and 17.9 μA in terms of ring-averaged values, and high- and low-current bands alternate repeatedly along the radial direction. These fluctuations should not be interpreted as evidence that a decreasing trend is absent. Only the innermost ring shows a modest decline, and the colour field is visibly patchier than on the inner surface, with band boundaries that are irregular rather than concentric. It should be noted that the Cu/steel galvanic current cannot be directly converted into the absolute corrosion rate of steel. In this work, galvanic current is used to characterize the temporal variation of galvanic corrosion activity at the coupling interface rather than to quantify the uniform or pitting corrosion rate. For the contour plot, the color scale was capped at 18 μA for visualization purposes rather than hardware saturation of the measurement equipment. The stable signal around 17.8 μA under intermittently wetted conditions is attributed to the persistent electrolyte film formed on the rusted surface. Based on 720 hourly means per surface, the Spearman correlations with time were −0.911 and −0.558 for the inner and outer surfaces, respectively. The corresponding linear slopes were −0.01853 and −0.00711 μA d^−1^, with block-bootstrap 95% confidence intervals of [−0.02239, −0.01473] and [−0.01138, −0.00286] μA d^−1^. Thus, the outer-surface fluctuations should not be interpreted as “no decay.” Both records showed an overall decreasing tendency with temporal fluctuations. These current trends alone do not establish absolute corrosion rates or a specific rust-growth mechanism.

## 4. Discussion

The two rust architectures of Figure 3 follow from the wetting regimes. The permanently immersed inner surface grows its rust under buffered temperature and transport-limited oxygen supply, which yields a thin, compact layer. According to the literature, the intermittently wetted outer surface undergoes repeated hydration, dehydration, and redox cycling between γ-FeOOH and Fe_3_O_4_ [7,8]. We emphasize that these crystalline phases are not directly identified in our work due to the lack of XRD/Raman measurement. Thickness and quality rank in opposite directions, and the following analysis quantifies the consequences. Both rusted surfaces are mixed electrodes obeying

(1) $$i=i_{\mathrm{corr}}\left\{\exp\left[\frac{2.303\left(E-E_{\mathrm{corr}}\right)}{\beta_{a}}\right]-\exp\left[\frac{-2.303\left(E-E_{\mathrm{corr}}\right)}{\beta_{c}}\right]\right\}$$

Linearising Equation (1) about *E*_corr_ gives the Stern-Geary relation, while impedance supplies the polarisation resistance as the sum of the interfacial terms of Figure 5c,

(2) $$R_{p}=\frac{B}{i_{\mathrm{corr}}}\;\;B=\frac{\beta_{a}\beta_{c}}{2.303\left(\beta_{a}+\beta_{c}\right)}$$

*R*_p_ = *R*_f_ + *R*_ct_ (3)

The polarization analysis gave apparent corrosion-current densities of 32.23 and 41.21 μA cm^−2^ for the inner and outer surfaces, respectively. The corresponding DC polarization resistances were 453.0 and 485.3 Ω cm^2^, whereas the impedance-derived values were 155.4 and 145.1 Ω cm^2^. Thus, the two resistance estimates differ in magnitude and ranking. Because the SternGeary coefficients also differ between the surfaces, resistance ranking alone cannot be directly equated with corrosion-rate ranking. Agreement among quantities derived from the same polarization fit is not independent validation. The present measurements do not determine the causes of the DC–EIS discrepancy, and the laboratory estimates should not be interpreted directly as service corrosion rates. Treating the rust as a porous conductor [17],

(4) $$R_{f}=\rho_{\mathrm{eff}}L\rho_{\mathrm{eff}}=\rho_{\mathrm{sol}}\frac{\tau }{\varepsilon }=\rho_{\mathrm{sol}}\varepsilon^{-m}$$

and forming the ratio between the surfaces so that *ρ*_sol_ cancels.

(5) $$\frac{R_{f,\mathrm{in}}}{R_{f,\mathrm{out}}}=\frac{L_{\mathrm{in}}}{L_{\mathrm{out}}}{\left(\frac{\varepsilon_{\mathrm{out}}}{\varepsilon_{\mathrm{in}}}\right)}^{m}$$

The lower fitted rust-layer resistance of the outer surface is consistent with its cracked and delaminated structure. However, uncertainties in effective electrochemical area limit quantitative interpretation of the resistance ratios and prevent a reliable determination of the connected-porosity ratio. A possible effect of the active-area fraction is illustrated by Equation (6).

(6) $$i_{a}=\frac{i_{\mathrm{corr}}}{f}f=\frac{A_{a}}{A}$$

Across the four measured fields per surface, the mean outer rust thickness was approximately three times the inner value. This suggests that flowing water strips loose rust from the inner surface, so thickness cannot be used to compare cumulative metal loss. Testing this needs wall-thickness data. It should be noted that all specimens originate from a single retired penstock section. Corrosion behaviour of penstock steel can be modulated by local flow vortices, weld seams, and local condensation micro-zones in real-world pipelines. Therefore, the findings from this component should be generalized to other penstock projects with caution. The conclusions rest on one component, one reference electrolyte, truncated impedance arcs, 720 h of monitoring, and 90 pits, too few for an extreme-value analysis of the tail [26,27]. Pit measurements may complement wall-thickness surveys, but broader sampling is needed before general inspection recommendations can be made.

## 5. Conclusions

The inner and outer surfaces of a service-retired Q355C hydropower penstock were compared on a single component using rust-layer cross-sectioning, electrochemical measurement, three-dimensional damage metrology, and 720 h of continuous galvanic-current monitoring. The following conclusions are drawn.

1. The examined surfaces differed in rust-layer morphology. The fitted charge-transfer resistance point estimates were close, at 140.7Ω·cm^2^ and 138.9Ω·cm^2^ for the inner and outer surfaces, respectively, but their interpretation is limited by fitting uncertainty and low-frequency extrapolation. These values do not establish identical intrinsic charge-transfer kinetics. The outer rust layer exhibited cracks and delamination. Within the fitted equivalent circuit, the rust-layer resistance accounted for 4.3% of the total interfacial resistance on the outer surface and 9.5% on the inner surface. Rust thickness and protectiveness therefore rank in opposite directions. The thickness comparison describes retained rust and may be affected by unquantified rust removal, including possible flow-induced stripping.
2. The polarization measurements yielded a higher apparent corrosion-current density for the outer surface under the laboratory test conditions. However, differences between the DC and impedance-derived polarization resistances limit their quantitative comparison, and these results do not establish differences in service corrosion rates or demonstrate rate equivalence. The outer-surface sample contained one high-K pit that strongly influenced its skewness and kurtosis. The proportions of pits with K > 0.5 did not differ significantly between the surfaces. These findings require cautious interpretation because of the limited sample size and sensitivity to a single extreme observation. Among the measured pits, none of the 59 inner-surface pits and one of the 31 outer-surface pits exceeded K = 0.5. This limited sample does not establish the extreme-pit behaviour of the entire penstock.
3. Both surfaces showed overall decreases in galvanic current over the available 720 h period. These observations alone do not establish the absence of an outer-surface decreasing trend or confirm a specific rust-growth mechanism. Pit measurements should be considered alongside wall-thickness data, with the present findings limited by the small pit sample.

## Figures and Tables

**Table 1:** Chemical composition of the Q355C penstock steel in wt%.

C	Si	Mn	P	S	Al(t)	Cr	Ni	Cu	Ti	Fe
0.18	0.20	1.35	0.012	0.014	0.039	0.02	0.01	0.02	0.02	Bal.

**Table 2:** Statistics of rust-layer thickness.

Surface	Number of Measured Fields	Mean Rust Thickness (μm)	Standard Deviation (μm)	Range of Thickness (μm)
Outer	4	147.91	16.56	127.73–167.21
Inner	4	49.38	24.12	28.73–74.53

**Table 3:** Polarisation parameters of the rusted specimens in 3.5 wt.% NaCl.

Specimen	*E*_corr_ (V vs. SCE)	*β*_a_ (mV dec^−1^)	*β*_c_ (mV dec^−1^)	*B* (mV)	*i*_corr_ (μA cm^−2^)	*R*_p_ (Ω cm^2^)
Inner surface	−0.643 ± 0.005	63.6 ± 2.1	71.3 ± 3.17	14.60 ± 0.56	32.23 ± 1.031	453.0 ± 17.39
Outer surface	−0.690 ± 0.0072	84.5 ± 4.3	101.2 ± 10.16	20.00 ± 1.47	41.21 ± 1.204	485.3 ± 35.76

**Table 4:** Equivalent-circuit parameters fitted to the impedance spectra of Figure 5.

Parameter	Inner Surface	95% Confidence Interval	Outer Surface	95% Confidence Interval
*R*_s_ (Ω cm^2^)	49.7	[48.77, 50.64]	19.7	[19.01, 20.40]
*Q*_f_ × 10^−2^ (S s^n^ cm^−2^)	1.19	[0.88, 1.49]	0.38	[0.25, 0.50]
*n*_f_	0.39	[0.31, 0.46]	0.570	[0.46, 0.68]
*R*_f_ (Ω cm^2^)	14.8	[10.78, 18.81]	6.21	[4.26, 8.16]
*Q*_dl_ × 10^−2^ (S s^n^ cm^−2^)	3.31	[2.77, 3.85]	4.46	[4.06, 4.86]
*n*_dl_	0.54	[0.43, 0.65]	0.48	[0.45, 0.51]
*R*_ct_ (Ω cm^2^)	140.7	[118.78, 162.62]	138.9	[121.87, 155.93]
*χ*^2^× 10^−5^	2.9	[2.16, 3.64]	7.72	[5.09, 10.35]
|*Z*| at 10 mHz (Ω cm^2^)	111.5	/	76.9	/
*R*_f_ + *R*_ct_ (Ω cm^2^)	155.4	/	145.1	/
*R*_f_/*R*_ct_	0.105	/	0.045	/

**Table 5:** Damage descriptors and pit shape factor statistics of the descaled surfaces.

Descriptor	Inner Surface	Outer Surface
*S*_a_ (μm)	11.05	23.98
Maximum pit depth (μm)	65.48	80.44
Number of pits *n*	59	31
Mean *K*	0.250	0.295
Standard deviation of *K*	0.101	0.134
Median *K*	0.234	0.294
Maximum *K*	0.493	0.831
Skewness	+0.61	+1.93
Kurtosis	−0.35	+6.35
Pits with *K* > 0.5 (%)	0	3.2

**Table 6:** Skewness and excess kurtosis of pit shape factor K with bootstrap 95% confidence intervals and sensitivity analysis.

Dataset	Pit Count	Skewness	95% CI of Skewness	Excess Kurtosis	95% CI of Excess Kurtosis
Inner surface	59	0.61	[0.20, 1.02]	−0.35	[−1.01, 0.73]
Outer surface	31	1.93	[−0.54, 2.55]	6.35	[−1.27, 9.32]
Outer surface, exclusion of K = 0.83	30	−0.18	[−0.67, 0.32]	−0.92	[−1.36, −0.05]

## Data Availability

Data will be made available on request.

## Supplementary Materials

The following supporting information can be downloaded at: https://www.mdpi.com/article/10.3390/ma19214616/s1, Table S1: Field service information of the sampled Q355C penstock specimen; Table S2: Water chemistry parameters.
