# Supplementary material for: Rust-Layer Characteristics and Pit Morphology on the Inner and Outer Surfaces of a Retired Q355C Hydropower Penstock
Source: Materials (Basel). 2026 Oct 30;19(21):4616. doi: 10.3390/ma19214616 (PMC13764913; doi:10.3390/ma19214616)
Supplement: Supplementary file 1 [file materials-19-04616-s001.zip › materials-4615146-supplementary.pdf]

Supplementary Information

Table S1 summarizes the on-site service background of the Q355C steel specimen cut from the hydropower penstock, including service life, coating failure history, sampling position, distance to weld seam and underground gallery environmental conditions. Table S2 lists the chemical composition of circulating water in the gallery. The steel surface was under intermittently wetted conditions induced by water splashing and vapor condensation, which matches the corrosion environment investigated in this work.

Table S1. Field service information of the sampled Q355C penstock specimen

Parameter	Value
Service duration	16 years of continuous operation after commissioning
Coating and breach history	Original coating: epoxy coal tar pitch coating. Local cracking and delamination appeared after ~11 years service. Sampling zone is within natural coating failure area, bare steel exposed to circulating water. No repair or re-coating was carried out on this region before sampling.
Sampling location	320 m upstream of turbine inlet
Distance to the nearest weld	480 mm away from circumferential weld, outside weld heat-affected zone.
Gallery temperature	14–18 °C
Gallery relative humidity	82–96% RH

Table S2. Water chemistry parameters

**Water chemistry index**	**Range**
pH	7.4–8.1
Dissolved oxygen, DO	6.2–8.5 mg/L
Electrical conductivity	210–330 μS/cm
Ca^{2+}	42–61 mg/L
Mg^{2+}	13–22 mg/L
HCO_3^-	145–190 mg/L
Cl^-	11–19 mg/L
